# Upregulated expression level of the growth factor, progranulin, is associated with the development of primary Sjögren’s syndrome

**DOI:** 10.3892/etm.2014.1981

**Published:** 2014-09-22

**Authors:** NI ZHANG, NING YANG, QILIN CHEN, FENG QIU, XINGFU LI

**Affiliations:** 1 Department of Hematology, Second Hospital of Shandong University, Jinan, Shandong 250033, P.R. China; 2 Center for Oncology, Provincial Hospital Affiliated to Shandong University, Jinan, Shandong 250021, P.R. China; 3 Department of Rheumatology, Qilu Hospital, Shandong University, Jinan, Shandong 250012, P.R. China

**Keywords:** primary Sjögren’s syndrome, progranulin, interleukin-6

## Abstract

The aim of the present study was to investigate the expression and effect of progranulin (PGRN) in patients with primary Sjögren’s syndrome (pSS). In total, 26 newly diagnosed pSS patients and 26 healthy subjects were enrolled in this study. The serum levels of PGRN and the inflammatory factor, interleukin-6 (IL-6), were detected using ELISA. In addition, the mRNA expression levels of these molecules were detected by quantitative polymerase chain reaction. The serum levels of PGRN and IL-6 in the pSS patients increased significantly compared with the healthy controls (P<0.05). During the remission stages, the levels of PGRN and IL-6 were comparable to those of the healthy controls. The serum level of PGRN in the pSS patients was shown to correlate with that of IL-6 in the pre-treatment and post-treatment stages. PGRN was upregulated in the pSS patients, indicating a possible role of PGRN in the pathogenesis and development of pSS.

## Introduction

Sjögren’s syndrome is a common chronic autoimmune disease characterized by lymphocytic infiltration of glands and ocular and oral dryness, which primarily affects the salivary and lacrimal glands. This syndrome may occur as a primary Sjögren’s syndrome (pSS), or in association with other systemic autoimmune diseases, such as rheumatoid arthritis and systemic lupus erythematosus (1). Sjögren’s syndrome may manifest within a wide spectrum of diseases, ranging from a limited, organ-specific autoimmune exocrinopathy to a systemic disease with widespread autoimmune manifestations and pronounced immunological features (2). pSS is characterized by polyclonal B cell activation, leading to chronic hypergammaglobulinemia, increased levels of β_2_-microglobulinemia and the concomitant presence of a variety of autoantibodies (3). Multiple factors, including viral infection, hormonal balance and genetic background, are involved in the pathogenesis of pSS. The presence of T and B cells, macrophages and dendritic cells varies according to the severity of the lesion (4). The influence of abnormal cytokine production in this disease has attracted considerable attention (5).

Progranulin (PGRN) is an autocrine growth factor with multiple physiological and pathological functions. PGRN can bind to tumor necrosis factor receptors and is therapeutic against inflammatory arthritis in mice (6). Therefore, PGRN is a potential target for the treatment of autoimmune diseases. However, the changes in PGRN expression in pSS patients remain unclear. In the present study, the serum levels of PGRN in the peripheral blood of pSS patients and healthy controls were examined to investigate the possible role of PGRN in the pathogenesis and development of pSS.

## Materials and methods

### Patients

In total, 26 newly diagnosed pSS patients were recruited for the study. All patients met the criteria revised by American College of Rheumatology in 1997 for the classification of pSS (7). None of the patients had been treated with immunosuppressive drugs prior to specimen collection. The patients received symptomatic and supportive treatment, as well as immunosuppressive therapy, within a period of 21 consecutive days. Peripheral blood samples were collected from the patients. The control group included 26 healthy volunteers, matching the gender and ages of the pSS patients (female, 25; male, 1; age range, 24–65 years; median age, 44.8±10.96 years). All the subjects signed informed consent forms prior to entering the study. Ethical approval for the research was obtained from the Medical Ethical Committee of Qilu Hospital, Shandong University (Jinan, China).

### ELISA

Coagulated blood (5 ml) was collected from each patient and control subject prior to and following the administration of prednisone. The blood was centrifuged (5000 × g for 10 min at 4°C) and the serum specimens were stored at −80°C. The serum levels of PGRN and the inflammatory factor, interleukin-6 (IL-6), were measured using a commercial ELISA kit (Yonghui Company, Beijing, China), according to the manufacturer’s instructions.

### Quantitative polymerase chain reaction (qPCR)

Peripheral blood mononuclear cells were separated using red blood cell lysis buffer (Pharmacia Diagnostics, Uppsala, Sweden) and the total RNA was isolated using TRIzol reagent (Invitrogen Life Technologies, Carlsbad, CA, USA), according to the manufacturer’s instructions. An Eppendorf Biophotometer (Brinkmann Instruments, Westbury, NY, USA) was used to determine the RNA concentration, and the concentration was adjusted to 1 μg/ml for reverse transcription. The RNA was reverse-transcribed to form cDNA using a ReverTra Ace qPCR RT kit (Toyobo Corporation, Osaka, Japan). qPCR was performed using the Light Cycler *Taq*Man Master kit (Toyobo Corporation), according to the manufacturer’s instructions, on a Bio-Rad IQ5 detection system (Bio-Rad Laboratories, Hercules, CA, USA). Fluorescence qPCR was performed using SYBR Green (Toyobo Corporation). Each sample was determined in triplicate, and the qPCR products were run on agarose gels to confirm the expected size of the samples. Melting-curve analysis was also performed to ensure the specificity of the products. The relative mRNA expression levels of IL-6 were determined using the comparative Ct method, using arithmetic formulae from the relative expression software tool (Bio-Rad Laboratories). The relative expression of PGRN was calculated using the ^ΔΔ^Ct method. The expression of mRNA was normalized against the expression of the GAPDH gene.

### Immunoblot analysis

Total proteins were harvested from the blood collected from the patients and control group. The proteins were separated using 10% SDS/PAGE, and subjected to immunoblot analyses. The primary mouse anti-human PGRN monoclonal antibody (clone 296628) was purchased from R&D Systems (Minneapolis, MN, USA), while the primary mouse anti-GAPDH monoclonal and secondary horseradish peroxidase-conjugated goat anti-mouse antibodies were purchased from Santa Cruz Biotechnology, Inc. (Santa Cruz, CA, USA). Bound antibodies were quantified using an enhanced chemiluminescence system (Pierce Biotechnology, Inc., Rockford, IL, USA). The experiments were performed three times.

### Statistical analysis

Statistical analysis was performed using SPSS 17.0 software (SPSS, Inc., Chicago, IL, USA). The data are presented as the median ± interquartile range and were analyzed with the Mann-Whitney U test. Comparisons among the pre-treated, post-treated and control groups were performed with an independent sample non-parametric test. In addition, correlations between PGRN and IL-6 levels were assessed using Spearman’s rank correlation. P<0.05 was considered to indicate a statistically significant difference.

## Results

### mRNA and protein expression levels of PGRN are increased in pSS patients

In this study, 26 newly diagnosed pSS patients were enrolled (Table I). The control group included 26 healthy volunteers, matching the gender and ages of the pSS patients. Among the pSS patients (Table I), 25 were female and one was male, with an age range of 24–65 years (median age, 44.8±10.96 years). The course of the disease from the initial appearance of symptoms to the enrollment in the study varied between 2 and 98 months (median disease course, 20.4±22.0 months).

**Figure 1:**
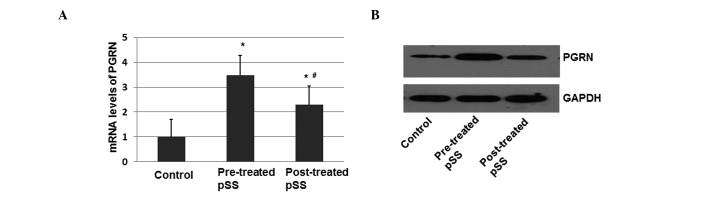
Quantitative polymerase chain reaction (qPCR) and immunoblot analysis. Peripheral blood mononuclear cells were separated from 26 healthy controls and 26 pSS patients prior to and following treatment with prednisone, and the total mRNA and protein were isolated. (A) Expression levels of PGRN mRNA were examined by qPCR and normalized against the expression of the GAPDH gene. (B) Protein expression levels of PGRN were examined by immunoblotting, where GAPDH was used as the loading control. Each sample was determined in triplicate and a representative blot is shown for one of the 26 patients. ^*^P<0.05, vs. healthy controls; ^#^P<0.05, vs. pre-treated pSS group. pSS, primary Sjögren’s syndrome; PGRN, progranulin.

To determine the mRNA expression level of PGRN, peripheral blood mononuclear cells from the healthy controls and pSS patients were separated prior to and following treatment with prednisone. The total mRNA was isolated and the mRNA expression level of PGRN was investigated with qPCR. Using IQ5 software, the data are presented as the fold change in the gene expression normalized against GAPDH. As shown in Fig. 1A, there was a 3.45-fold increase in the relative mRNA expression of PGRN in the pSS patients prior to treatment with prednisone (10 mg) when compared with the healthy controls (P<0.05; Fig. 1A). Following treatment with prednisone, the mRNA expression level of PGRN showed only a 1.6-fold increase when compared with the healthy controls (P<0.05). The difference in the expression levels before and after treatment with prednisone was statistically significant (P<0.05).

In order to determine the protein expression levels of PGRN, peripheral blood mononuclear cells were separated and the total proteins were isolated and determined by immunoblotting. The protein expression level of PGRN was increased in the pSS patients prior to treatment when compared with the healthy controls (P<0.05; Fig. 1B). Following treatment, the protein expression levels of PGRN decreased (Fig. 1B). These results indicated that PGRN expression may be positively associated with the development of pSS.

### Serum PGRN levels are increased in pSS patients

ELISA was performed to investigate the serum levels of PGRN (Table II), and IL-6 served as the cytokine control. As demonstrated in Table II, the levels of PGRN in pSS patients were significantly upregulated when compared with the healthy controls (P<0.05; Table II). In addition, the difference between the PGRN levels prior to and following prednisone treatment was statistically significant (P<0.05). Following treatment, the serum levels of PGRN were significantly downregulated, but remained higher than the healthy control levels (P<0.05; Table II). The IL-6 levels were higher in pSS patients prior to treatment when compared with the healthy control (P<0.05) and post-treatment patient groups (P<0.05; Table II). Therefore, the PGRN level in the patients was altered based on the development of pSS.

### PGRN levels correlate with IL-6 in pSS patients

**Figure 2:**
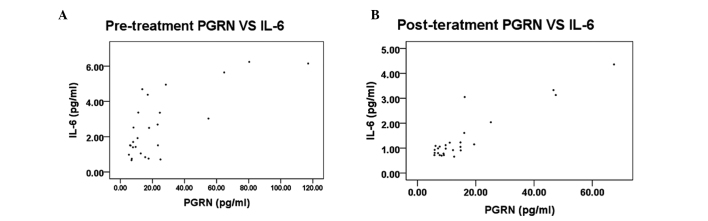
Spearman’s rank correlation analyses of PGRN and IL-6 levels in pSS patients (A) prior to and (B) following treatment with prednisone. P<0.05 indicates a statistically significant difference. PGRN, progranulin; IL-6, interleukin-6; pSS, primary Sjögren’s syndrome.

To examine the association between the serum levels of PGRN and the pSS-related inflammatory factor, IL-6, Spearman’s rank correlation analysis was performed in pSS patients prior to and following treatment. The results demonstrated that the serum level of PGRN in the pre-treatment group correlated with the level of IL-6 (r=0.617, P=0.001; Fig. 2A). Similarly, the serum level of PGRN in the post-treatment group correlated with the level of IL-6 (Fig. 2B).

## Discussion

PGRN is an autocrine growth factor containing 7.5 repeats of a cysteine-rich motif in the order, P-G-F-B-A-C-D-E, where P is the half motif (8). PGRN is predominantly expressed in epithelial and immune cells, neurons (9) and chondrocytes (10), and high expression levels of PGRN are found in a variety of human cancer types (11). Several studies have revealed that PGRN plays an important role in a number of pathological processes, including early embryonic development, wound healing and inflammation (12–17). PGRN also functions as a regulator of cartilage development and degradation (18). PGRN, binding directly to the tumor necrosis factor receptor, is involved in a number of physiological and pathological functions. Upregulation of PGRN has been reported in chemotherapy-induced amenorrhea (19).

The present study demonstrated that the levels of PGRN in the peripheral blood were upregulated in the pre-treated and post-treated pSS patients when compared with the healthy controls, indicating that PGRN may be involved in the development of pSS. In the pre-treated pSS patients, the levels of IL-6 were higher compared with the control and post-treated patient groups. In addition, the IL-6 levels were shown to linearly correlate with the levels of PGRN (P<0.05). IL-6 has been identified as an important factor in the pathogenesis of pSS (20), and murine lupus models have demonstrated the involvement of IL-6 in B-cell hyperactivation and the onset of systemic lupus erythematosus (21,22).

In conclusion, the present study demonstrated that PGRN is upregulated in pSS patients, indicating a possible role of PGRN in the pathogenesis and development of pSS.

## Figures and Tables

**Table I:** Clinical parameters of the pSS patients.

Patients	Gender	Age (years)	Disease course (months)	ESR (mm/h)	RF	anti-SSA Ab
1	F	48	71	115/76	+	+
2	F	35	2	9/15	+	+
3	F	32	26	67/87	−	−
4	F	46	22	31/15	+	+
5	M	51	98	57/39	+	+
6	F	33	19	22/16	+	+
7	F	43	33	11/7	+	+
8	F	51	7	52/46	+	+
9	F	52	8	63/31	−	−
10	F	58	29	15/9	+	+
11	F	63	39	19/25	−	+
12	F	48	15	24/11	+	+
13	F	65	28	46/25	+	+
14	F	56	8	75/49	−	+
15	F	34	6	16/8	+	+
16	F	26	2	69/49	+	+
17	F	39	3	29/12	−	−
18	F	46	9	78/48	+	+
19	F	35	10	33/27	−	−
20	F	45	2	23/16	+	+
21	F	24	4	56/20	+	+
22	F	36	6	39/25	−	−
23	F	45	26	69/33	+	+
24	F	42	22	85/30	+	+
25	F	55	20	39/26	−	−
26	F	58	16	37/21	+	+

pSS, primary Sjögren’s syndrome; F, female; M, male; ESR, erythrocyte sedimentation rate; RF, rheumatoid factor; anti-SSA Ab, anti-Sjögren’s syndrome A antibody.

**Table II:** Comparison of serum levels of PGRN and IL-6 by ELISA.

Groups	PGRN (pg/l)	IL-6 (pg/ml)
pSS		
Pre-treatment	14.57±7.93^a^^b^	1.81±1.03^a^^b^
Post-treatment	10.39±7.47^a^	1.05±079
Healthy control	9.80±5.67	0.84±0.69

a P<0.05, vs. healthy control and

b P<0.05, vs. post-treatment pSS group.

IL-6, interleukin-6; pSS, primary Sjögren’s syndrome.
